# Child Care Time, Parents’ Well-Being, and Gender: Evidence from the American Time Use Survey

**DOI:** 10.1007/s10826-016-0416-7

**Published:** 2016-04-07

**Authors:** Anne Roeters, Pablo Gracia

**Affiliations:** Utrecht University, Utrecht, The Netherlands; The Netherlands Institute of Social Research, PO Box 16164, 2500 BD The Hague, The Netherlands; European University Institute, Florence, Italy

**Keywords:** Parental care time, Gender, Time use, Wellbeing, Multi-level methods

## Abstract

This study used data from the ‘Well Being Module’ of the 2010 American Time Use Survey (N = 1699) to analyze how parents experience child care time in terms of meaning and stress levels. Multivariate multilevel regressions showed clear differences by gender and the circumstances of child care activities. Mothers experienced child care time as more stressful than fathers, and fathers as slightly more meaningful. Interactive child care was experienced as more meaningful and less stressful than routine child care, whereas these differences were stronger among fathers than among mothers. Mothers experienced child care with a minor child as highly meaningful, and with an adolescent as particularly stressful. Fathers experienced child care with an infant as highly stressful, and with an offspring in middle childhood as disproportionally meaningful. The spouse’s presence was moderately associated with higher senses of meaning and lower levels of stress during child care, but these differences were modest, and only visible among fathers. Paid work hours increased mothers’ stress levels during child care activities, but reduced fathers’ stress levels. Meanwhile, nonemployed fathers reported child care time as less meaningful than non-employed mothers. Overall, this study has important scientific and practical implications for `understanding the gendered nature of parents’ child care time and well-being.

## Introduction

Studying how parents experience child care time is critical to understand parents’ socio-emotional wellbeing. Parental care time, and particularly the quality of parenting activities, plays an important role in promoting child well-being (Gryczkowski et al. 2010; Hsin and Felfe 2014; Sandberg and Hofferth 2001). But parent–child time can also influence parents’ wellbeing. Parents tend to value their time with children positively, because it promotes family solidarity and has a high intrinsic value (Bianchi et al. 2006). Yet, parents’ child care time might also have negative consequences for their wellbeing, given that parenting practices are known to be highly demanding and time consuming activities (Crouter et al. 2004; Perry-Jenkins et al. 2000).

Previous studies found that parents generally experience high levels of well-being when spending time in child care (Brown et al. 2008; Musick et al. 2013; Offer 2014; Offer and Schneider 2011). The type of child care activity was found to play an important role in parents’ wellbeing. Offer’s (2014) study with American data on dual-earner middle-class couples found that parents enjoy interactive child care (i.e., playing with children) to a higher extent than other child care activities, such as routine child care (i.e., physical care). Offer (2014) also found that mothers disproportionally engage in routine child care, which captures gender inequalities in the division of labor and parents’ wellbeing. These studies offer relevant information to understand how parents experience child care time.

The literature suggests that parents’ experience of parent–child time may be affected by other characteristics of the parent and activity as well. The spouse’s presence in the child care activity, for example, is likely to play an important role. We know that mothers, and especially fathers, spend an important proportion of their child care time in shared activities with the spouse (Craig 2006). Given that the partner is a source of instrumental and emotional support, it is likely that the spouse’s presence during parenting activities increases the perceived quality of child care (Roeters and Treas 2011). Another critical aspect is children’s developmental stages. Parent–child attachment and relations change dramatically across children’s developmental stage, affecting parents’ energy and effort put into child care (Gracia 2014; Nomaguchi and Milkie 2003; Phares et al. 2009). Thus, parents’ well-being experienced during child care time might differ depending on children’s developmental stages. Although the literature suggests that parents’ well-being may not be only affected by the type of activity, but also by the specific circumstances of parent–child time, previous studies offered little evidence on how the specific circumstances of parents’ child care time influence parents’ well-being.

Two aspects of parents’ well-being during parent–child time are especially interesting to study: ‘meaning levels’ and ‘stress levels.’ Because prior research shows that individuals can experience both positive and negative emotions during the same activity (Kahneman et al. 2004), it is relevant to study both positive and negative affect. The sense of meaning during child care time captures whether parents perceive child care as important for them and for children, and whether participating in this activity is in line with their (role) expectations. Stress levels, on the other hand, reflect the perceived demands of child care. These two indicators are particularly interesting from a gender perspective. Women tend to feel more responsible for child care and emotionally attached to their children than men (Hays 1996), which may increase women’s meaning levels experienced during child care, but also their perceived stress. Overall, analyzing the factors that bring parents to experience child care with higher or lower levels of meaning and stress is relevant for the literature on parenting and parent’s emotional wellbeing.

The remaining part of this Introduction elaborates upon five possible determinants of parents’ subjective experience of child care that so far received limited empirical attention: (1) gender; (2) the type of activity; (3) the presence of the partner; (4) children’s developmental stages; (5) paid work characteristics.

First, *gender* is a central variable. Gender inequalities in parenting are strong, as mothers are still the main providers of child care, especially in the most demanding activities (Bianchi et al. 2006; Craig 2006; Esping-Andersen et al. 2013). The traditional view that women hold the main responsibility for child care persists (Kotila et al. 2013) and parenting norms still portray the mother–child bond as central for child development, while fathers are often perceived as secondary child care providers (Rizzo et al. 2012). Mothers display stronger role expectations than fathers when caring for children, and were found to enjoy child care to a higher extent than fathers (Grote et al. 2004; Kotila et al. 2013; Poortman and Van der Lippe 2009). Also, mother–child time is particularly demanding, because it is more frequently combined with other domestic work activities (Mattingly and Bianchi 2003; Offer 2014) and because mothers have higher childrearing standards than fathers fathers (Poortman and Van der Lippe 2009). This suggests that mothers might experience child care as more meaningful and stressful than fathers.

Second, the type of *child care activity* is likely to influence how parents experience child care time. Routine child care activities (e.g., feeding children, putting children to bed and supervising children) are typically perceived as more demanding and less enjoyable than interactive child care activities (i.e., socializing and playing with children) (Bianchi et al. 2006; Craig 2006; Gracia and Esping-Andersen 2015; Roeters et al. 2010). As a result, parents may experience interactive child care as more meaningful and less stressful than routine child care. Research on dual-earner middle-class couples from the United States found that parents experience higher levels of well-being when participating in interactive child care, as compared to other child care activities (Offer 2014). Yet, to our knowledge, evidence for the general population in this direction is lacking so far. Because fathers are less inclined than mothers to protect female-typed child care (i.e., physical care), and to value this time as a central component of their identity (Hays 1996), it is likely that fathers disproportionally experience low meaning levels and high stress levels when engaging in routine child care.

Third, the role of the *spouse* is relevant to understand how parents experience child care time. Parents are highly motivated to participate in child care activities with the spouse, because this promotes family socializing and bonding (Daly 2001; Glorieux et al. 2011; Gracia and Kalmijn 2015). The presence of the spouse can make activities more enjoyable and less demanding, given that spouses can interact and share tasks (Roeters and Treas 2011). Prior research suggests that fathers and mothers attach different meanings to sharing child care time with the spouse. Fathers disproportionally engage in child care with the spouse, and are more likely than mothers to receive active support from the spouse during the couple’s shared child care (Craig 2006). Fathers frequently demand the spouse’s help in child care, partly because mothers assign fathers the role of secondary child care providers (Hays 1996). Altogether, it is plausible that fathers experience child care activities with the spouse as more meaningful and less stressful than mothers.

Fourth, *children’sdevelopmental stages* is arguably an important predictor of how parents experience child care time. Parents may experience their time with minor children as especially meaningful, because the benefits and satisfaction associated with a recent entry into parenthood are high (Nomaguchi 2012; Umberson et al. 2010). Nevertheless, given that babies and toddlers require particularly high levels of attention, and often produce parental fatigue and tiredness (Shanahan et al. 2007; Umberson et al. 2010), time with very young children is likely to be highly stressful. Also, parents may experience high levels of stress in their time with adolescents, compared to their time with children in middle childhood. This is likely to be the case because parent–child conflicts with adolescents are more frequent than with children in middle childhood, and parents can more frequently suffer distance or relationship problems with adolescents (Laursen et al. 1998). Prior research suggests that the impact of children’s age on the experience of time is different for fathers and mothers. Mothers tend to have a strong emotional attachment to the child during the early years of life, whereas parenting norms reinforce the “essential” role of mothering during early childhood (Rizzo et al. 2012). Mothers invest more effort and energy than fathers in caring for minor children, especially in the most demanding physical activities (Gracia 2014), which would have consequences on stress levels perceived during child care time with a minor child.

Fifth, *paid work characteristics* are likely to be important determinants of how parents experience child care. The ‘enrichment approach’ suggests that employed individuals are better prepared than non-employed individuals to enjoy and successfully engage in different life domains (Greenhaus and Powell 2006). Parents who engage in paid work develop skills and gain resources that benefit their role as parents, having potential positive implications for family life and mental health (Hofferth and Goldscheider 2010; Danner-Vlaardingerbroek et al. 2013; Perry-Jenkins et al. 2000). Thus, being employed is likely to be linked to high meaning levels and low stress levels experienced during child care. However, as paid work hours increase, child care time may also be experienced more negatively. The ‘conflict approach’ posits that parents who work longer hours lack the energy and attention that is needed to enjoy child care, given that time, energy, and attention are scarce resources (Greenhaus and Powell 2006; Roeters et al. 2010). Therefore, working longer hours may be associated with lower meaning levels (e.g., being too tired to be responsive to children) and higher stress levels (e.g., feeling guilty or hurried) during parent–child time. Gender is likely to be important in how paid work is associated with parents’ experience of child care. Mothers feel more responsible for child care than fathers do (DeVault 2000; Hays 1996), while this sense of responsibility is difficult to reconcile with paid work constraints, arguably resulting in feelings of guilt, conflict, and stress (Craig and Mullan 2013; Nomaguchi and Milkie 2003). This suggests that increasing working hours can result in lower levels of meaning and higher levels of stress among mothers than among fathers.

This study uses data from the ‘Well Being Module’ of the *2010 American Time Use Survey* (ATUS) to analyze how parents experience child care time by analyzing two questions: (1) how meaningful child care time is to parents; (2) how stressful child care time is experienced by parents. We estimated Multivariate Multilevel Models, allowing us to account for the possible correlation between these two outcomes (Hox 2012). Drawing on the literature, we develop different hypotheses. *Hypothesis 1* states that mothers experience parental care time as more meaningful and more stressful than fathers. *Hypothesis 2* anticipates that parents experience interactive child care as more meaningful and less stressful than they experience routine child care time, with stronger differences for fathers than for mothers. *Hypothesis 3* expects child care time with the spouse to be experienced as more meaningful and less stressful than solo child care time, with stronger differences for fathers than for mothers. *Hypothesis 4a* predicts that parents experience child care time with minor children as more meaningful than with older children. *Hypothesis 4b* anticipates that parents experience child care time with minor children and adolescents with higher levels of stress than they experience child care time with children in their mid childhood. *Hypothesis 4c* expects the effects of child care time with minor children on meaning and stress levels to be stronger for mothers than for fathers. *Hypothesis 5a* states that parents who participate in employment experience parent–child time more meaningfully and less stressfully than parents who do not participate in employment. Finally, *Hypothesis 5b* anticipates that, as parents’ paid work hours increase, they experience parental care time as less meaningful and more stressful, and that these effects are stronger among mothers than they are among fathers.

## Method

### Participants

We used the *2010 American Time Use Survey* (ATUS), which contains a stratified random sample of the American population with excellent data to analyze the relationship between parents’ time use and their emotional states (Lee et al. 2015). Data were collected from January through December 2010, with a response rate of 57 %. The ATUS measured respondents’ time use through computer-assisted telephone interviewing (CATI). The ‘Daily Reconstruction Method’ (DRM) was applied. The DRM captures specific emotional states by asking respondents to recall their feelings for each selected activity (Kahneman et al. 2004). Respondents were asked to reconstruct their time use on the day before the interview. Half of respondents were assigned a random weekday, and the other half were assigned a random weekend day (U.S. Bureau of Labor Statistics 2014). The ‘Well Being Module’ of the 2010 ATUS randomly selected three activities from the completed time diary, asking different questions on how these activities were experienced.

### Procedures

We selected those respondents who reported having at least one minor biological child in the home. Other households were excluded because parents’ interactions with stepchildren or non-resident children differ substantially from those with biological children (Amato 2004), which could generate bias in our estimates. We selected episodes from the ‘Well Being Module’ in which parents reported conducting a primary child care activity (codes 030101 through 030399), ranging from interactive care, physical care, and parental supervision, among other activities. We excluded from our sample of analysis those respondents with missing values on this measure. This selection procedure resulted in a sample consisting of a maximum of three episodes per respondent in a single day. The final sample had 1699 episodes nested in 1378 parents. Mothers were overrepresented (1170 episodes; 935 respondents), given that mothers spend more time in child care than fathers do (U.S. Bureau of Labor Statistics 2014).

### Measures

#### Dependent Variables

We used two dependent variables on the experience of parental care time. ‘Meaning levels’ was measured with the following question: “From 0 to 6, how meaningful did you consider what you were doing?”. ‘Stress levels’ was based on this question: “From 0 to 6, how stressful did you consider what you were doing?”. Values on these one-item measures ranged from 0 (not at all) to 6 (very much).

#### Independent Variables

We used several independent variables. One dummy variable differentiated between ‘interactive care’ (reading to or with the child; indoor/outdoor play; talking with or listening to the child; teaching/educational care) and ‘routine care’ (parental supervision; physical-related care) (0 = non-interactive; 1 = interactive). We also used a dummy for the ‘spouse’s presence’ during the parental care activity (0 = no; 1 = yes). Additionally, we constructed a categorical variable of the ‘child’s developmental stage’, looking at the age of the youngest child present in the activity: ‘babies’ (aged 0–1), ‘toddlers’ (aged 2–3), ‘middle childhood children’ (aged 4–11), and ‘adolescents’ (aged 12–17). Young children receive more parental time and attention than older children (Bittman and Wajcman 2000). Thus, we chose the age of the youngest child present as a way to identify the potential effect of children’s developmental stages on the experience of parents’ child care time. Finally, two measures of paid work were used, namely the respondent’s ‘employment status’ (0 = non-employed; 1 = employed) and ‘weekly paid work hours’ (ranging from 0 to 70 h). Non-employed respondents were assigned a ‘0’ on the measure for work hours.

#### Controls

We included several control variables in the analyses. At the episode level, we controlled for the duration of the parenting activity episode (in minutes), and also for when the activity took place during the day (0 = not night; 1 = night). Longer episodes provide more opportunities for meaningful ‘quality time’, whereas parent–child time during the night is likely to be stressful. At the respondent level, we controlled for ‘parental education’, one critical predictor of parenting (Bianchi et al. 2006), which had three categories: (1) high-school degree or below, (2) basic college education; (3) BA degree or higher. We also controlled for ‘marital status’, namely whether the respondent was single or lived with a partner (0 = cohabiting or married; 1 = single parent), which can condition parental care time and time poverty (Monna and Gauthier 2008). We considered the ‘number of children’ at home, a continuous measure that can account for parental care demands and is typically included in the literature (Bianchi et al. 2006). We used a dummy variable of ‘race’, a variable that can influence parents’ time use and parenting styles (Hofferth 2003; Monna and Gauthier 2008) (0 = white; 1 = non-white). Finally, we considered if the activity occurred on a weekend (0 = no, 1 = yes), as parents’ time availability affect their child care time (Bianchi et al. 2006).

### Analysis Plan

The analysis strategy followed different steps. First, descriptive analyses, *t* tests and *χ*^2^ tests, were used to describe the sample, and to test the basic gender differences in the subjective experience of parental care time. Second, Multivariate Multilevel Analyses (MMA) were conducted. Because the episodes are nested within individuals, multilevel models are appropriate for these purposes. MMA models estimated the regressions for meaning levels and stress levels simultaneously. This approach accounts for the correlation between our two outcomes, minimizing bias and gaining statistical power in a multivariate statistical framework (Hox 2012). Although multilevel models are highly robust, we used robust standard errors (Maas and Hox 2004). Models were estimated using MLWiN (Rasbash et al. 2005). Given that there were some discrepancies between the *p*-values with the Wald test and the Chi square test, the latter test was used (Hox 2012: 45). We first run analyses separately by gender and, subsequently, pooled the sample of fathers and mothers to run gender interactions for all variables.

## Results

Table 1 reports the weighted descriptive statistics of our measures of analysis, as well as the results of *t* tests and * χ*^2^ tests on gender differences for each measure. Fathers and mothers reported, respectively, 5.36 and 5.26 on the measures of meaning levels, and 1.13 and 1.20 in the scale of stress. The average for meaning is that high because two-third of the parents rated the activity with the highest score. Even though gender differences are modest, the *t* test reveals significant differences at the 90 % level of confidence for meaning levels, and of 95 % for stress levels. The correlations between the scale of stress and meaning (not shown) were −0.16 for men, and −0.08 for women. This implies that the two measures are negatively associated, but also that this association is relatively moderate.

**Table 1:** Descriptive statistics

	Fathers		Mothers		Range	Gender differences^a^
	*M/p*	*SD*	*M/p*	*SD*		*p* value
*Episode level*	N = 529		N = 1170			
Meaning levels	5.36	1.28	5.26	1.48	0–6	0.09^†^
Stress levels	1.13	1.50	1.21	1.64	0–6	0.02*
Youngest child present						
Baby	0.12		0.20		0–1	0.00***
Toddler	0.41		0.45		0–1	0.18
Mid childhood	0.43		0.29		0–1	0.06^†^
Adolescence	0.04		0.06		0–1	0.50
Spouse’s presence	0.38		0.16		0–1	0.00***
Interactive child care	0.55		0.40		0–1	0.00***
Night episode	0,02		0,03		0–1	0.11
Episode’s length (min)	96,92	104,53	71,01	64,06	10–760	0.00***
*Respondent level*	*N* = 443		*N* = 935			
Non-employed	0.19		0.49		0–1	0.00***
Weekly work hours	33.95	20.18	16.26	18.88	0–70	0.00***
Education						
High school or lower	0.37		0.36		0–1	0.15
Basic college education	0.24		0.26		0–1	0.07*
BA levels or higher	0.39		0.38		0–1	0.02^**^
Single-parent family	0.04		0.22		0–1	0.00***
Number of children	2.26	0.97	2.11	1.21	1–10	0.29
Non white	0.15		0.19		0–1	0.02**
Weekend diary	0.33		0.27		0–1	0.17

^a^ *p* value based on two-sided *t* test or *χ* ^2^ test: *** *p* < 0.001; ** *p* < 0.01; * *p* < 0.05; ^†^ *p* < 0.10

The independent variables and controls show interesting variations. At the episode level, a lower proportion of child care episodes took place with a baby among fathers (12 %), as compared to mothers (20 %) (*p* < 0.001). By contrast, fathers were more likely to report the presence of a child in mid childhood (43 %) than mothers did (29 %) (*p* < 0.10). Also, a higher proportion of child care episodes with the spouse was found among fathers (38 %) than among mothers (16 %), even if these gender differences were statistically insignificant. We see that the proportion of child care episodes with interactive activities was higher for fathers (55 %) than it was for mothers (40 %), with significant gender differences (*p* < 0.001). We also observe that < 4 % of the episodes occurred during the night, for both men and women. Finally, we observe that the average minutes of child care episodes were higher among fathers (97) than among mothers (71) (*p* < 0.001).

At the respondent level, we found relevant descriptive differences, both within and between genders. We observe that 19 % of fathers reported not being employed when the interview took place, compared to 49 % of women being out of the labor force (*p* < 0.001). These differences are higher than the average maternal employment rate in the United States, as a result of the overrepresentation of non-employed women in the sample (U.S. Bureau of Labor Statistics 2014). The mean weekly paid work hours was 34 for fathers and 16 h for mothers, and these gender differences were significant (*p* < 0.001). We observe similar educational levels for mothers and fathers, with most parents reporting ‘BA levels or higher’ and ‘High school or lower’. Mothers were more likely to be single parents (22 %) than fathers (4 %). Also, a lower proportion of fathers was classified as non-white (15 %), as compared to mothers (19 %). Finally, the proportion of individuals reporting child care time in the weekend was slightly higher for fathers (33 %) than for mothers (27 %).

Tables 2 and 3 present the results of the MMA models with independent and control variables. Before testing our hypotheses on meaning and stress, we ran the empty models (without independent variables) to calculate the intra-class coefficient (ICC). The ICC’s for fathers were 63 % for meaning and 63 % for stress levels. For mothers, the ICC’s were 55 % for meaning and 63 % for stress. This means that between half and two-thirds of the variance in meaning and stress levels lies on the respondent level.

**Table 2:** Multivariate multilevel models explaining parents’ meaning levels experienced during parent–child time

	Fathers		Mothers		Gender effects^a^
	*B*	*SE*	*B*	*SE*	*p* value
*Episode level*					
Independent variables					
Interactive child care	0.58	0.11***	0.21	0.09*	0.13
Spouse’s presence	0.22	0.10^†^	0.03	0.10	0.20
Youngest child present					
Baby (aged 0–1)	−0.32	0.20	0.30	0.12*	0.02*
Toddler (aged 2–3)	−0.11	0.13	0.03	0.09	0.18
Mid childhood (aged 4–11)	(Ref)		(Ref)		(Ref)
Adolescence (aged 12–17)	−0.71	0.23**	0.02	0.17	0.04*
Controls					
Night episode	0.13	0.56	0.61	0.26*	0.31
Length of episode (min)	0.00	0.00	0,00	0.00***	0.06^†^
*Respondent level*					
Independent variables					
Employed	0.45	0.28	0.16	0.16	0.04*
Weekly work hours	0.00	0.01	0.00	0.00	0.18
Controls					
Education					
High school or lower	0.20	0.17	−0.20	0.11^†^	0.13
Basic college	(Ref)	(Ref)	(Ref)		
BA levels or higher	−0.20	0.14	−0.19	0.10^†^	0.19
Single-parent family	0.11	0.25	0.19	0.11	0.40
Number of children	−0.22	0.07***	−0.10	0.11	0.04*
Non white	0.36	0.19^†^	−0.07	0.09	0.17
Weekend diary	−0.04	0.12	−0.02	0.04	0.30
*Constant*	5.57	0.28***	5.34	0.20***	
−2/LL	3514.831		8332.772		

N_Fathers_ = 529 observations from 443 respondents/N_Mothers_ = 1170 observations from 935 respondents

*** *p* < 0.001; ** *p* < 0.01; * *p* < 0.05; ^†^ *p* < 0.10; We used two-sided tests for the main effects, and one-sided tests for the gender interactions

^a^Gender interactions based on a pooled sample of fathers and mothers

**Table 3:** Multivariate multilevel models explaining parents’ stress levels experienced during parent–child time

	Fathers		Mothers		Gender effects^a^
	*B*	*SE*	*B*	*SE*	*p* value
*Episode level*					
Independent variables					
Interactive child care	−0.50	0.13***	−0.30	0.11**	0.06^†^
Spouse’s presence	−0.09	0.13	−0.05	0.13	0.07^†^
Youngest child present					
Baby (aged 0–1)	0.42	0.22^†^	−0.46	0.16**	0.08^†^
Toddler (aged 2–3)	0.19	0.14	−0.17	0.12^†^	0.06^†^
Mid childhood (aged 4–11)	(Ref)		(Ref)		(Ref)
Adolescence (aged 12–17)	0.38	0.31	0.86	0.22***	0.09^†^
Controls					
Night episode	−0.34	0.54	0.18	0.32	0.32
Length of episode (min)	0.00	0.00	0.00	0.00	0.00***
*Respondent level*					
Independent variables					
Employed	−0.02	0.32	0.20	−0.21	0.11
Weekly work hours	−0.01	0.01*	0.01	0.01**	0.01**
Controls					
Education					
High school or lower	−0.65	0.19***	−0.23	0.15	0.15
Basic college	(Ref)		(Ref)		(Ref)
BA levels or higher	−0.07	0.17	−0.09	0.14	0.14
Single-parent family	0.56	0.29^†^	0.21	0.14	0.14
Number of children	0.13	0.07^†^	0.15	0.23	0.15
Non white	−0.02	0.20	−0.15	0.15	0.11
Weekend diary	−0.29	0.15^†^	0.13	0.05*	0.06^†^
*Constant*	1.27	0.32***	1.21	0.26***	
−2/LL	3522.831		8332.772		

N_Fathers_ = 529 observations from 443 respondents/N_Mothers_ = 1170 observations from 935 respondents

*** *p* < 0.001; ** *p* < 0.01; * *p* < 0.05; ^†^ *p* < 0.10; We used two-sided tests for the main effects, and one-sided tests for the gender interactions

^a^Gender interactions based on a pooled sample of fathers and mothers

Table 2 presents the MMA on meaning levels. Fathers rated child care activities as more meaningful if the spouse was present, although these effects were quite moderate (*p* < 0.10). Also, fathers rated parent–child time as less meaningful when the youngest child present was an adolescent, as compared to parental care time in which a child in middle childhood was the youngest child present (*p* < 0.01). We observe that fathers experienced their time in interactive child care as more meaningful than their time in routine child care (*p* < 0.001). Finally, employment participation and weekly paid work hours did not influence fathers’ meaning levels experienced during parental care time.

For mothers, the MMA results of Table 2 show that the spouse’s presence was not significantly associated with how meaningful child care was experienced. The developmental stage of the youngest child present had a different effect for mothers than for fathers. Mothers rated child care time with a baby as the youngest child present as more meaningful than the time with a child in middle childhood as the youngest child present (*p* < 0.05). There were no significant differences between mothers’ time with toddlers or adolescents and their time with children in middle childhood. Like for fathers, mothers’ experienced interactive child care as more meaningful than routine child care, yet these effects were weaker than for fathers (B_mothers_ = 0.21; B_fathers_ = 0.58) *p* < 0.05). Finally, for mothers’ paid work, we did not find significant differences in meaning levels linked to child care time.

Table 2 also shows the gender interactions for meaning levels experienced during parent–child time (last column). Surprisingly, the difference for interactive care is not significant, despite the considerable gender differences observed in the effect sizes. For the spouse’s presence, the gender interactions did not reveal any significant difference. Yet, we found relevant gender differences in other variables. For the developmental stage of the youngest child present, the interaction effects show that differences between mothers and fathers in meaning levels experienced during parental care time were significant for both the presence of babies and adolescents (*p* < 0.05). Finally, the results of gender interactions show that non-employed mothers experienced parental care time with lower meaning levels than non-employed fathers (*p* < 0.05).

Table 3 presents the MMA on stress levels experienced during parental care time for fathers and mothers. For fathers, we observe that they did not experience child care time with a partner as significantly more or less stressful. Fathers experienced child care with babies as more stressful than they did with offspring in middle childhood as the youngest child present (*p* < 0.10). Fathers rated parental care time with a toddler or adolescent as more stressful than their time with a child in middle childhood, but these differences were insignificant. Also, fathers clearly reported lower levels of stress during parental care time when they engaged in interactive child care activities (*p* < 0.001). Finally, fathers’ weekly work hours were associated with a reduction in their stress levels during child care activities (*p* < 0.01).

For mothers, Table 3 reveals that the spouse’s presence did not significantly affect their stress levels. Regarding the developmental stage of the child, mothers’ stress was significantly lower when a baby was present in the activity, compared to child care activities in which an offspring in mid childhood was the youngest present (*p* < 0.01). Spending time in child care activities with a toddler was moderately less stressful for mothers than spending time with an offspring in mid childhood as the youngest child present (*p* < 0.10). By contrast, mothers rated parental care time as more stressful when an adolescent was the youngest child present than when the youngest child present was in mid childhood (*p* < 0.001). Finally, we observe that mothers’ stress levels were higher when they worked longer hours (*p* < 0.01). This effect is opposite to the effect for fathers.

The gender interactions for stress levels during child care time can be found also in Table 3 (last column). Gender differences in stress levels experienced during child care were moderately significant for all the developmental stages of the child (*p* < 0.10). The gender interactions for the weekly paid work hours was significant, in line with the observed differences between fathers and mothers (*p* < 0.001). Finally, the negative effect of spending time in interactive child care on parents’ stress levels during child care time was stronger for fathers than it was for mothers (*p* < 0.10).

Finally, we summarize the main findings of the control variables of Tables 2 and 3. For fathers, the MMA show that parental care time was experienced less meaningfully as the number of children in the home increased, whereas fathers’ stress levels were much lower among low-educated fathers than among fathers with higher levels of education. Non-white fathers experienced higher levels of meaning during parental care time than white fathers, while being a single father increased the levels of stress during parental care time. We can also see that fathers experienced less stress during child care on weekends than on weekdays. For mothers, the control variables indicate lower levels of meaning experienced during parental care time for both mothers with the lowest and with the highest educational levels. Finally, unlike for fathers, mothers were found to experience higher levels of stress during child care time on weekends than on weekdays.

## Discussion

This study analyzed how mothers and fathers in the United States experience child care time. We investigated two central questions to understand the subjective experience of parent–child time: How meaningful child care time was for the parent and which levels of stress parents experienced during child care time. The ‘Well Being Module’ of the *2010 American Time Use Survey* (ATUS), a survey with a representative sample of American families, provided a unique possibility to investigate these questions. These data allowed us to provide a more global picture of how Americans experience their child care time than in previous related studies, which typically focused on more restricted samples, such as dual-earner middle-class couples (Offer 2014). Our study offered new evidence on how the specific circumstances of parenting activities, such as the presence of the spouse and developmental stage of the child present, influence the subjective experience of parent–child time.

We tested several hypotheses related to the circumstances of the child care activity, as well as parents’ characteristics. First, we hypothesized that the gendered nature of parenting brings mothers to experience child care as more meaningful, but also as more stressful, than fathers (*Hypothesis 1*). Results were partly consistent with this hypothesis. As expected, the average stress levels during parental care time were substantially higher for mothers than for fathers. This finding is in line with previous research suggesting that mothers invest more energy and emotional effort in demanding parenting activities than fathers, and also that mothers are more prone than fathers to combine child care with demanding domestic activities, which is likely to influence mothers’ stress (Offer 2014). Nevertheless, against expectations, fathers experienced child care time as slightly more meaningful than mothers did. This finding might reflect that fathers select into those activities that are more meaningful to them and are less energy demanding, while mothers’ child care time has a larger component of more repetitive routine activities (Gracia and Esping-Andersen 2015). This fact might bring mothers to comparatively attribute lower meaning levels to child care activities. Still, this result is somehow surprising, given that research on gender roles suggests that mothers rate parental child care as a more rewarding and importantactivity than fathers do (Grote et al. 2004; Kotila et al. 2013; Poortman and Van der Lippe 2009).

Second, we hypothesized that interactive child care is experienced as more meaningful and less stressful than routine child care, and also that these differences are stronger for fathers (*Hypothesis 2*). Results reveal that, indeed, parents experienced interactive child care as a more meaningful and less stressful activity than routine child care. Also, consistent with expectations, the positive effect of interactive child care on meaning levels, and negative effect of interactive child care on stress levels, was stronger for fathers than for mothers. Although gender interactions for the type of child care activity had moderate effects, and only for stress levels, results show that the difference in how interactive and routine child care is experienced is larger for fathers than for mothers. These results differ from the study of Offer (2014) on dual-earner middle-class couples, in which she found that mothers experience non-interactive child care as a more stressful activity than fathers. It might be the case that part of the differences between our study and Offer’s (2014) study is the result of the selectivity of her sample, which is composed of employed middle-class parents.

Third, we expected child care activities with the spouse to be experienced with higher meaning levels and lower stress levels than solo child care activities, and that the effects would be stronger for fathers than for mothers (*Hypothesis 3*). The empirical evidence was limited however. Parents experienced child care time with the spouse as more meaningful and less stressful than their solo child care, but these differences were insignificant. Only for fathers we found that the spouse’s presence increased meaning levels during child care time. The effect of the spouse’s presence was stronger for fathers than for mothers, but differences were moderate. These results complement previous qualitative studies on family time by offering a quantitative perspective (Daly 2001). They suggest that the spouse’s presence can benefit the subjective experience of parental care time, especially for fathers, but also that other factors play a more important role in how child care time is experienced.

Fourth, we formulated multiple expectations related to children’s developmental stages. We anticipated child care time with minor children to be experienced as more meaningful than with older children (*Hypothesis 4a*), and child care time with minor children or adolescents as more stressful than with children in mid childhood (*Hypothesis 4b*). We also expected mothers to experience child care with minor children with higher meaning and stress levels than fathers do (*Hypothesis 4c*). Results were generally consistent with expectations. For mothers, we indeed found high levels of meaning for their child care time when an infant was present, and mothers experienced child care time with an adolescent as more stressful than with an offspring in mid childhood. Yet, mothers’ stress levels during child care were lowest when an infant was present. In contrast, fathers experienced time with a child in middle childhood present as particularly meaningful, and time with infants as more stressful.

Empirical results regarding children’s developmental stages bring us to several analytical interpretations. Mothers’ low stress with infants can reflect a strong gendered attachment to the baby (Hays 1996). The existence of some risks of parent–child conflict with adolescents (Laursen et al. 1998), combined with mothers’ tendency to arrange children’s schooling and extracurricular activities for adolescents (Hays 1996), can explain the high levels of stress in mothers’ time with adolescents that were observed. These findings for mothers, however, need to be analyzed in future studies by looking at the specific child care activities in which mothers and fathers engage. Meanwhile, fathers’ negative evaluations of child care with infants, and positive evaluations of child care with children in middle childhood may reflect a lower attachment of fathers to preschoolers, as a result of a gendered organization of child care (Rizzo et al. 2012). These findings contribute to research on the gendered nature of parenting throughout the life course.

Fifth, we hypothesized that employed parents experience child care time more meaningfully and less stressfully than non-employed parents (*Hypothesis 5a*). Also, we anticipated that increasing work hours leads parents to experience child care time as less meaningful and more stressful and that these effects were stronger for mothers than for fathers (*Hypothesis 5b*). Being employed was associated to higher meaning levels during child care, but these effects were insignificant. Interestingly, we did find a significant interaction effect between gender and employment. The effect was stronger for mothers, which may reflect that stay-at- home mothers attach higher value to child care and are more satisfied with their role in the household division of labor than stay-at-home fathers. For paid work hours, we found opposite effects by gender, with fathers experiencing child care as less stressful as they increased their paid work hours and mothers as more stressful as they worked longer hours. These gendered outcomes resonate earlier findings implying that mothers experience more difficulties in combining work and family demands than fathers, given that they invest more energy and effort in child care time than fathers do, leading to fatigue and stress (Bianchi and Milkie 2010). Fathers who increase their hours of paid work may invest less energy or effort into child care activities, or evaluate child care as a less stressful activity compared to the high work demands that they encounter at the workplace. These findings reveal clear gender differences in terms of work-family balance and socio-emotional wellbeing.

We must acknowledge three limitations. First, our analyses included only those parents who participated in child care on the day of observation. This group might have less time constraints than people who did not engage in child care, limiting the generalization of our results. Still, we used a nationally representative sample of American families, unlike recent related studies with American data, which were restricted to a selected sample of the population (Offer 2014). Second, we only focused on episodes that conceptualized child care as a primary activity. Although studying primary child care is critical to understand how parents experience child care time, parents can engage in a primary activity combined with child care as a secondary activity (e.g., cooking while watching the child). Alternatively, the child could be present in one activity (e.g., leisure with children), even if the activity is not coded as child care. Future studies should offer a more multidimensional picture of parent–child time connected to parents’ wellbeing. Third, we unfortunately only had access to one diary, either on weekday or weekend. This impeded us to compare weekdays and weekends within the same parent, a relevant question to better understand the links between parenting and socio-emotional wellbeing.

Overall, our study offers novel evidence on the gendered nature of parents’ child care time and their socio-emotional wellbeing in the United States. Our results suggest that scholars need to analyze the specific conditions in which parenting activities occur to better understand the mechanisms that relate child care to parents’ emotional states in everyday life. Ideally, future studies should take into account additional factors that played only a secondary role in our study, such as family structure, social class, and ethnicity. Moreover, since parents’ sense of meaning during child care has seldom been studied, it would be interesting to further explore what this concept captures and how parents’ sense of meaning during child care compares to their experience of other activities. We leave the analysis of these aspects for future studies.

Finally, our study has important implications for professionals and policy makers. The finding that mothers and fathers differ in how they experience child care, and particularly depending on children’s life stages, is critical to understand the links between parenting and parents’ emotional wellbeing. Professionals and policy-makers in the field of work-family balance will be particularly interested in the gendered links that we found between paid work time and the experience of parental care time. Family-friendly and gender equality policies should address the fact that mothers disproportionally suffer work-family conflict, which reflects gender inequalities in employment careers, health, and individual wellbeing. We hope that our study contributes, not only to scientific debates on families and children, but also to professional and policy debates on these important issues.
